# Supplementary material for: Persistence of alveolar fibroblast-derived ADAMTS4+ cells in a preclinical model of delayed pulmonary fibrosis resolution
Source: Nat Commun. 2026 May 8;17:4205. doi: 10.1038/s41467-026-72419-3 (PMC13156320; doi:10.1038/s41467-026-72419-3)
Supplement: Supplementary file 1 — Supplementary Information [file 41467_2026_72419_MOESM1_ESM.pdf]

Supplementary material

Persistence of alveolar fibroblast-derived ADAMTS4+ cells in a preclinical model of delayed pulmonary fibrosis resolution

Mahsa Zabihi[1,2,3], Ali Khadim[1,2,3], Arun Lingampally[1,2,3,4*], Ana Ivonne Vazquez-Armendariz[5], Stefan Hadzic[2,3,4], Georgios-Dimitrios Panagiotidis[1,2,3,4], Daniel Kalina[2,3,4,6], Jan Halweg[1,2,3], Tara Procida-Kowalski[2,3,7], Marek Bartkuhn[2,3,7], Xuran Chu[8,9], Janine Koepke[2,3,4], Christos Samakovlis[2,3,4], Mario Boehm[6], Norbert Weissmann[2,3,4], Andreas Günther[2,3,4], Werner Seeger[2,3,4], Peter Braubach[10], Susanne Herold[1,2,3], Malgorzata Wygrecka[2,3,4,6], Saverio Bellusci[11,12*], Elie El Agha[1,2,3,8*]

[1]Department of Medicine V, Internal Medicine, Infectious Diseases and Infection Control, Universities of Giessen and Marburg Lung Center (UGMLC), German Center for Lung Research (DZL), German Center for Infection Research (DZIF), Justus-Liebig University Giessen (JLU), 35392 Giessen, Germany

[2]Cardio-Pulmonary Institute (CPI), 35392 Giessen, Germany

[3]Institute for Lung Health (ILH), 35392 Giessen, Germany

[4]Department of Medicine II, Internal Medicine, Pulmonary and Critical Care, Universities of Giessen and Marburg Lung Center (UGMLC), German Center for Lung Research (DZL), Justus-Liebig University Giessen, 35392 Giessen, Germany

[5] Transdisciplinary Research Area Life and Health, Organoid Biology, Life & Medical Sciences Institute, University of Bonn, 53115 Bonn, Germany

[6]CSL Innovation GmbH, 35041 Marburg, Germany

[7]Biomedical Informatics and Systems Medicine, Justus-Liebig University Giessen (JLU), 35392 Giessen, Germany

[8]Oujiang Laboratory (Zhejiang Lab for Regenerative Medicine, Vision and Brain Health), School of Pharmaceutical Science, Wenzhou Medical University, Wenzhou, Zhejiang 325035, China

[9]School of Pharmaceutical Sciences, Wenzhou Medical University, Wenzhou, Zhejiang 325035, China

[10]Institute for Pathology, Hannover Medical School, 30625 Hannover, Germany

[11]Department of Pulmonary and Critical Care Medicine, The Quzhou Affiliated Hospital of Wenzhou Medical University, Quzhou People's Hospital, Quzhou, Zhejiang 324000, China

[12]Laboratory of Extracellular Matrix and Regeneration, Justus-Liebig University Giessen (JLU), Cardio-Pulmonary Institute (CPI), German Center for Lung Research (DZL), Institute for Lung Health (ILH), 35392 Giessen, Germany

These authors contributed equally: Mahsa Zabihi, Ali Khadim

*Correspondence to: A.L. (e-mail: arun.lingampally@innere.med.uni-giessen.de), S.B. (e-mail: saverio.bellusci@innere.med.uni-giessen.de), or E.E.A. (e-mail: elie.el-agha@innere.med.uni-giessen.de)

Lead author: elie.el-agha@innere.med.uni-giessen.de

Supplementary figures

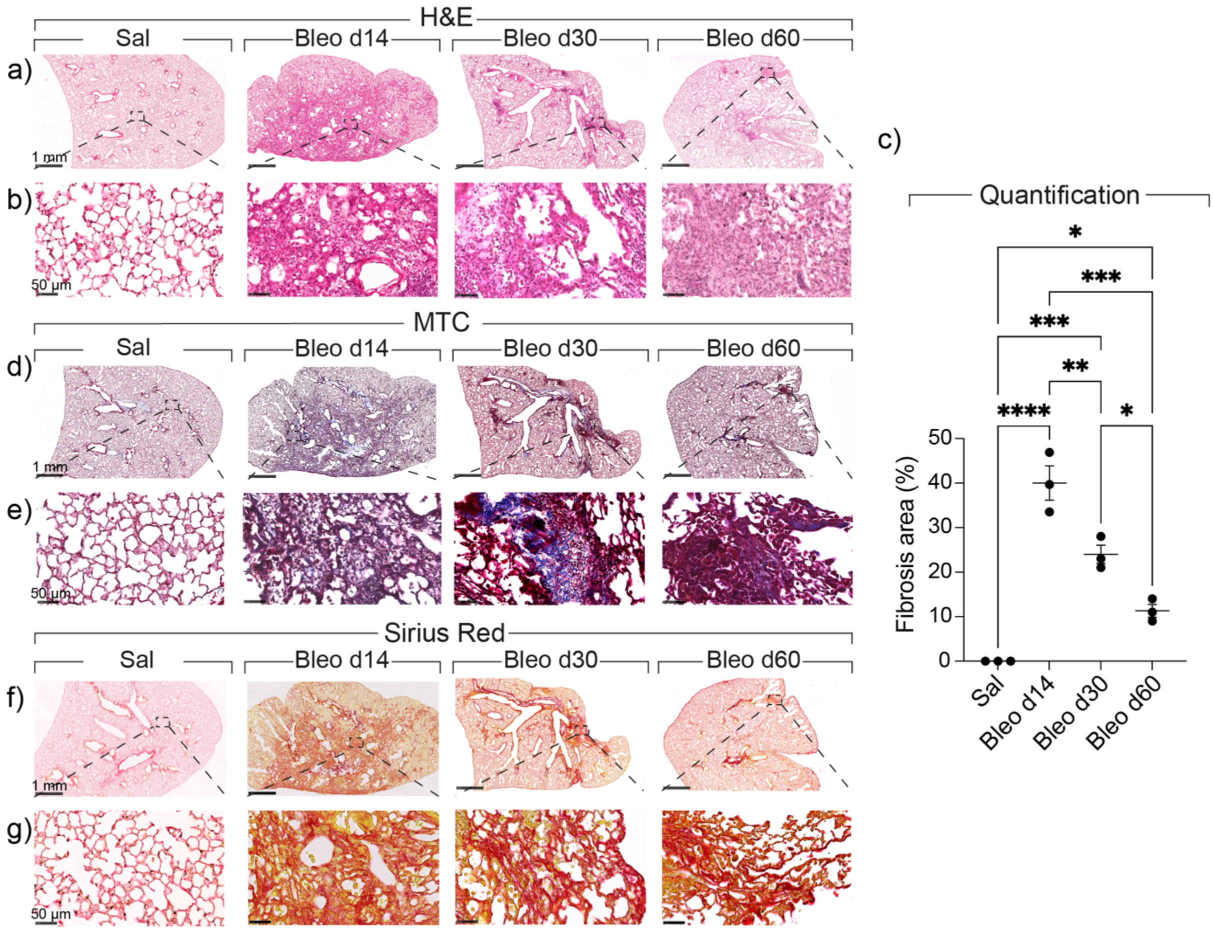

Supplementary Figure 1. Dynamics of fibrosis formation and resolution. a) Representative images of hematoxylin and eosin (H&E) stains of lung sections from saline- and bleomycin-treated groups at the indicated timepoints. b) High-magnification images of the dashed boxes shown in a). c) Quantification of lung damage according to H&E stains. d) Representative images of Masson's Trichrome (MTC) staining in lung sections from saline- and bleomycin-treated groups. Collagen fibers are stained blue. e) High-magnification images of the dashed boxes shown in d). f) Representative images of Picro Sirius Red staining in lung sections from saline- and bleomycin-treated groups. Collagen fibers are stained red. g) High-magnification images of the dashed boxes shown in f). n = 3 per group. Each data point represents one biological replicate. Scale bars: (a, d, f) 1 mm; (b, e, g) 50 µm. Data are presented as mean ± SEM. Statistical analysis was performed using ordinary one-way ANOVA with Tukey's multiple comparisons test. * $P < 0.05$; ** $P < 0.01$; *** $P < 0.001$; **** $P < 0.0001$. Bleo: Bleomycin; Sal: Saline.

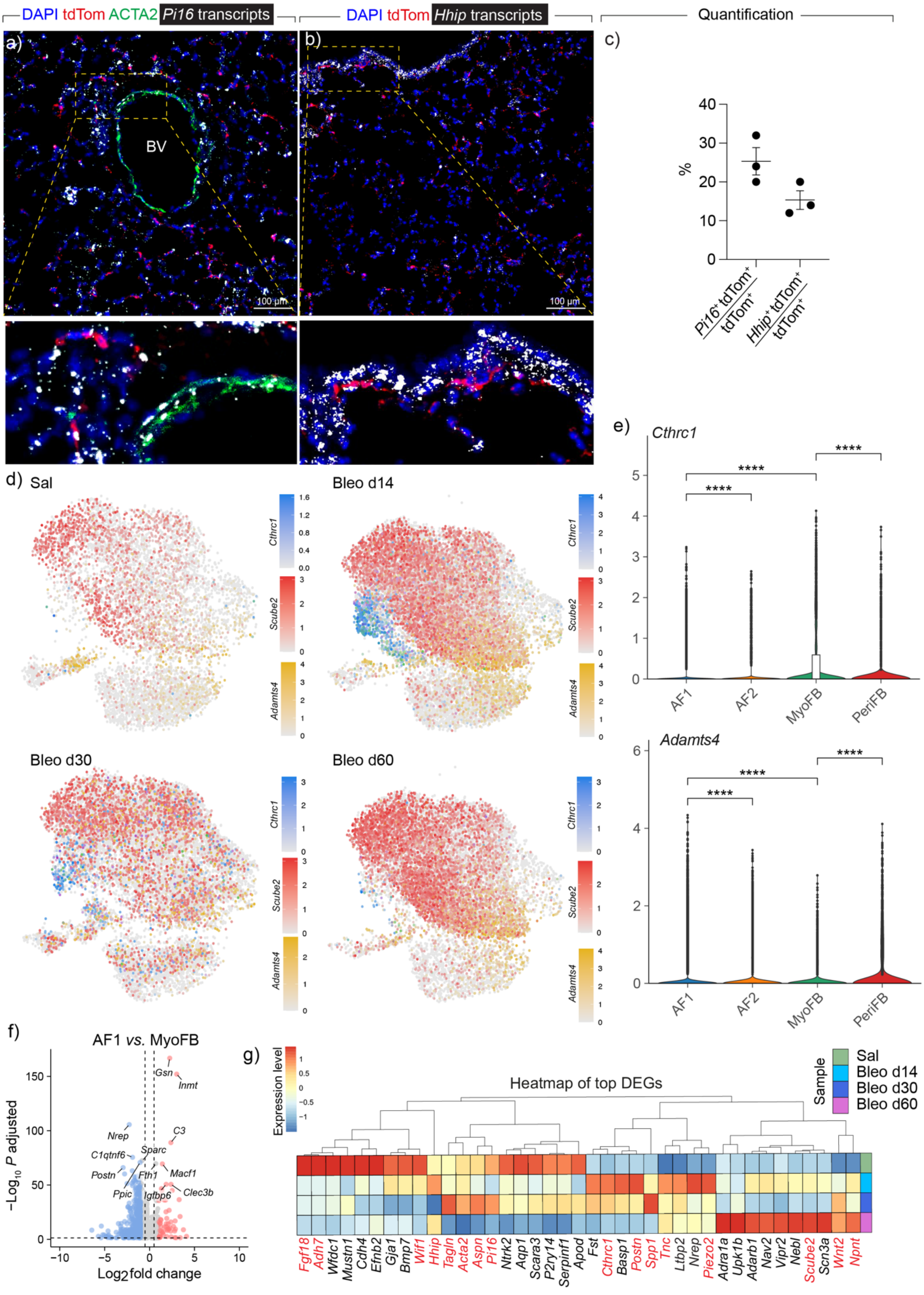

Supplementary Figure 2. Heterogeneity of the FGF10+ lineage. a, b) Representative images of in situ hybridization for *Pi16* or *Hhip* (white) and immunofluorescence for ACTA2 (green) and tdTom (red) in saline-treated *Fgf10*$^{Cre-}$

^{ERT2}*; tdTomato*^{flox} lungs. The dashed boxes are magnified in the lower panels. c) Quantification of the in situ hybridization and immunofluorescence data. d) Feature plots showing expression of indicated genes. e) Violin plots showing the expression levels of the indicated genes across different cellular clusters. f) Volcano plot showing top markers between AF1 and MyoFB. g) Heatmap of top DEGs in each condition. Expression level was calculated based on Log_2FC of the top 10 genes in each sample. Statistical analysis was performed using Wilcoxon signed-rank test. **** $P < 0.0001$. ACTA2: Actin alpha 2, smooth muscle; *Adamts4*: ADAM metallopeptidase with thrombospondin type 1 motif 4; AF1: Alveolar fibroblasts 1; Bleo: Bleomycin; BV: Blood vessel; *Cthrc1*: Collagen triple helix repeat containing 1; DAPI: 4′,6-diamidino-2-phenylindole; *Hhip*: Hedgehog interacting protein; MyoFB: Myofibroblasts; Sal: Saline; *Scube2*: Signal peptide, CUB domain and EGF like domain containing protein 2.

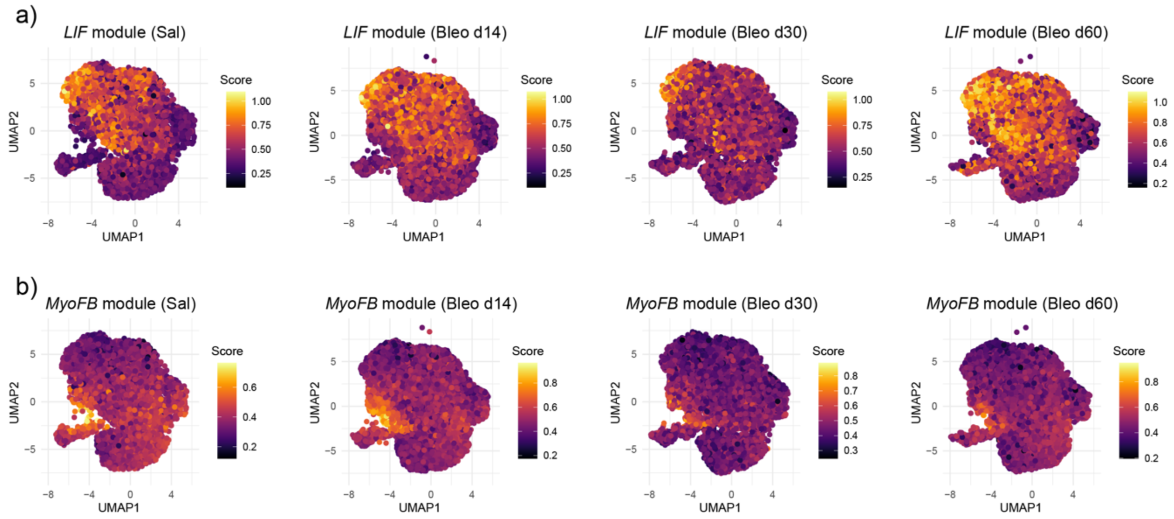

Supplementary Figure 3. Dynamics of lipofibroblast and myofibroblast signatures. a) UMAP plots showing the score of the LIF signature across samples. b) UMAP plots showing the score of the MYO signature across samples. Bleo: Bleomycin; LIF: Lipofibroblast; MyoFB: Myofibroblast; Sal: Saline.

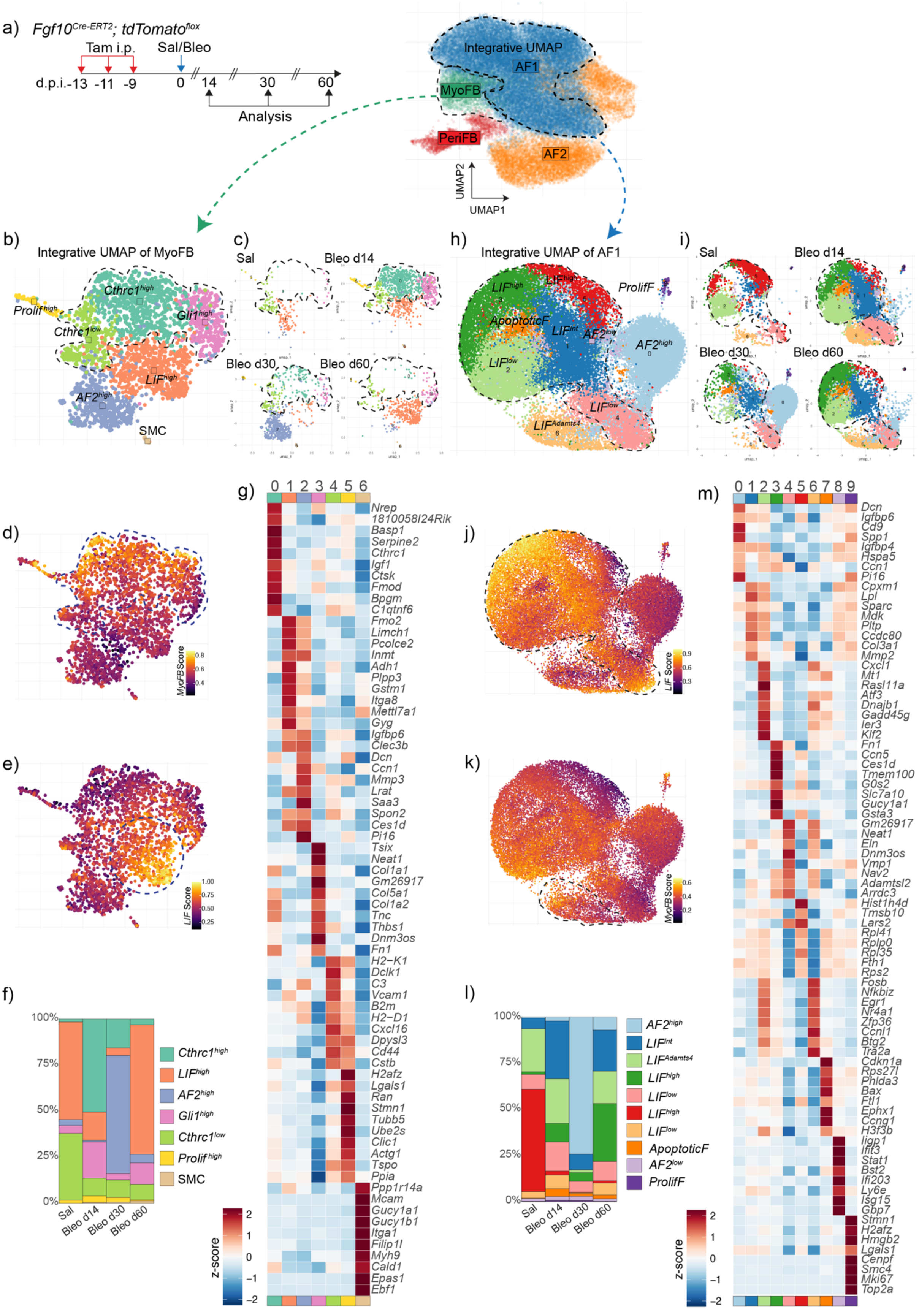

Supplementary Figure 4. Subclustering of myofibroblasts and lipofibroblasts. a) Timeline and schematic of the experimental design. b) Integrative UMAP of MyoFB

subclusters. c) UMAP plots of MyoFB subclusters in individual timepoints. d, e) UMAP plots showing the score of *MyoFB* and *LIF* signatures. f) Frequency of subclusters in each condition. g) Heatmap showing top genes across subclusters. h) Integrative UMAP of AF1 subclusters. i) UMAP plots of AF1 subclusters in individual timepoints. j, k) UMAP plots showing the score of *LIF* and *MyoFB* signatures. l) Frequency of subclusters in each condition. m) Heatmap showing top genes across subclusters. The dotted lines in b), c), and d) mark the subclusters with high *MyoFB* score and those in e), h), i), and j) mark the subclusters with high *LIF* score. The dotted line in k) highlights the MyoFB score in the $LIF^{Adamts4}$ subcluster. Source data are provided as a Source Data file. AF1: Alveolar fibroblasts 1; AF2: Alveolar fibroblasts 2; ApoptoticF: Apoptotic fibroblast; Bleo: Bleomycin; *Cthrc1*: Collagen triple helix repeat containing 1; LIF: Lipofibroblasts; MyoFB: Myofibroblasts; PeriFB: Peribronchial fibroblasts; Sal: Saline.

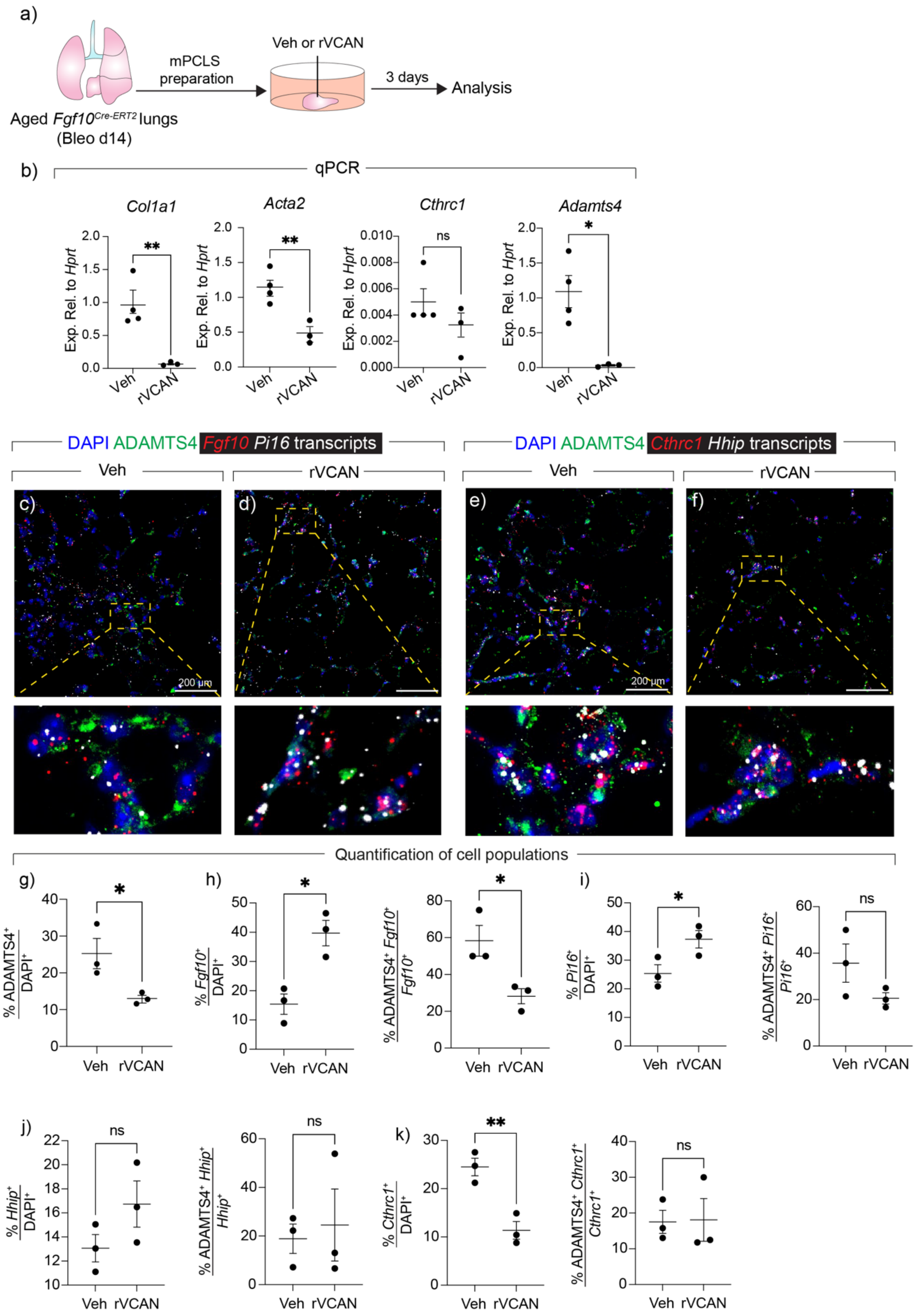

Supplementary Figure 5. Recombinant versican attenuates fibrosis in mouse precision-cut lung slice cultures. a) Timeline and schematic of the experimental design. b) Quantitative PCR for the indicated genes. c, d) Representative images of in situ hybridization for *Fgf10* (red) and *Pi16* (white) and immunofluorescence for

ADAMTS4 (green). The dashed boxes are magnified in the lower panels. e, f) Representative images of in situ hybridization for *Cthrc1* (red) and *Hhip* (white) and immunofluorescence for ADAMTS4 (green). The dashed boxes are magnified in the lower panels. (g-k) Quantification of the in situ hybridization and immunofluorescence data. b) n = 4 for Veh, n = 3 for rVCAN; g-k) n = 3 per group. Each data point represents one biological replicate. Data are presented as mean ± SEM. Statistical analysis was performed using student's t test (unpaired, two-tailed). * P < 0.05; ** P < 0.01; ns: Not significant. *Acta2*: Actin alpha 2, smooth muscle; ADAMTS4: ADAM metallopeptidase with thrombospondin type 1 motif 4; *Col1a1*: Collagen type I alpha 1 chain; *Cthrc1*: Collagen triple helix repeat containing 1; DAPI: 4′,6-diamidino-2-phenylindole; *Fgf10*: Fibroblast growth factor 10; *Hhip*: Hedgehog interacting protein; mPCLS: Mouse precision-cut lung slices; *Pi16*: Peptidase inhibitor 16; rVCAN: Recombinant versican; Veh: Vehicle.

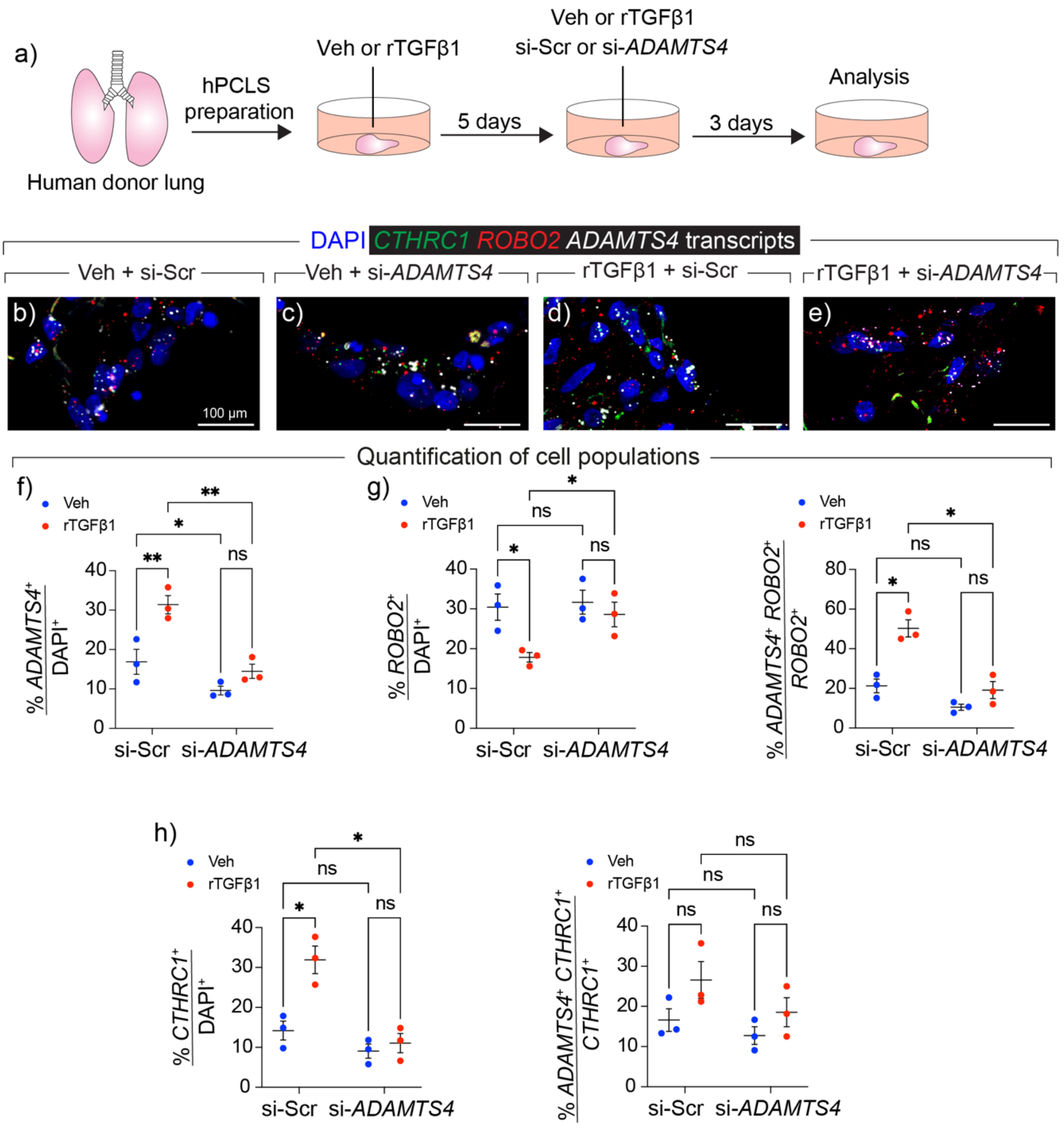

Supplementary Figure 6. Silencing *ADAMTS4* attenuates fibrogenesis in human precision-cut lung slice cultures. a) Timeline and schematic of the experimental design. b-e) Representative images of in situ hybridization for *CTHRC1* (green), *ROBO2* (red), and *ADAMTS4* (white) in different experimental groups. f-h) Quantification of the in situ hybridization data. n = 3 per group. Each data point represents one biological replicate. Data are presented as mean ± SEM. Statistical analysis was performed using RM two-way ANOVA with multiple comparisons. * $P < 0.05$; ** $P < 0.01$; ns: Not significant. *ADAMTS4*: ADAM metallopeptidase with thrombospondin type 1 motif 4; *CTHRC1*: Collagen triple helix repeat containing 1; DAPI: 4′,6-diamidino-2-phenylindole; hPCLS: Human precision-cut lung slices; *ROBO2*: Roundabout guidance receptor 2; rTGFβ1: recombinant transforming growth factor beta 1; si-*ADAMTS4*: siRNA targeting *ADAMTS4*; si-Scr: Scrambled siRNA; Veh: Vehicle.
