# Supplementary material for: Persistence of alveolar fibroblast-derived ADAMTS4+ cells in a preclinical model of delayed pulmonary fibrosis resolution
Source: Nat Commun. 2026 May 8;17:4205. doi: 10.1038/s41467-026-72419-3 (PMC13156320; doi:10.1038/s41467-026-72419-3)
Supplement: Supplementary file 2 — Description of Additional Supplementary Files [file 41467_2026_72419_MOESM2_ESM.pdf]

Description of Additional Supplementary Files

Supplementary Data 1: Human and mouse primer sequences used for quantitative PCR.
