# Supplementary material for: Persistence of alveolar fibroblast-derived ADAMTS4+ cells in a preclinical model of delayed pulmonary fibrosis resolution
Source: Nat Commun. 2026 May 8;17:4205. doi: 10.1038/s41467-026-72419-3 (PMC13156320; doi:10.1038/s41467-026-72419-3)
Supplement: Supplementary file 3 — Supplementary Data 1 [file 41467_2026_72419_MOESM3_ESM.docx]

**Supplementary Table 1: Human and mouse primer sequences used for quantitative PCR.**

| Gene | Forward primer (5´- 3´) | Reverse primer (5´- 3´) |
| --- | --- | --- |
| *hACTA2* | CTGTTCCAGCCATCCTTCAT | TCATGATGCTGTTGTAGGTGGT |
| *hADAMTS4* | TCACTGACTTCCTGGACAATGGC | GGTCAGCATCATAGTCCTTGCC |
| *hCEBPB* | AGAAGACCGTGGACAAGCACAG | CTCCAGGACCTTGTGCTGCGT |
| *hCOL1A1* | ATGTTCAGCTTTGTGGACCTC | CTGTACGCAGGTGATTGGTG |
| *hCTHRC1* | CAGGACCTCTTCCCATTGAAGC | GCAACATCCACTAATCCAGCACC |
| *hHOPX* | ATTCCACCACGCTGTGCCTCAT | AGTCTGTGACGGATCTGCACTC |
| *hHPRT* | AAGGACCCCACGAAGTGTTG | GGCTTTGTATTTTGCTTTTCCA |
| *hINMT* | ATTCGCCTGTGAGCTGGAAGGA | AGGTGGACATCGCACTTGAGCA |
| *hLIMCH1* | CTGGAGCTGAAACAAGACAACGG | GTCCTCTTGACTATTCGGTTCCG |
| *hNPNT* | CAGGTGCATGAACACTTACGGC | CACAGCCATACTGACAGTTTGCC |
| *hPBGD* | TGTCTGGTAACGGCAATGCG | CCCACGCGAATCACTCTCAT |
| *hPECAM1* | AAGTGGAGTCCAGCCGCATATC | ATGGAGCAGGACAGGTTCAGTC |
| *hPDGFRA* | CCACCTGAGTGAGATTGT | TCTTCAGGAAGTCCAGGT |
| *hPLIN2* | TCAGCTCCATTCTACTGTTCACC | CCTGAATTTTCTGATTGGCAC |
| *hPPARG* | TCTGCAAACATATCACAAGAAATGAC | ATATCAAAGGAGTGGGAGTGG |
| *hSFTPC* | GTCCTCATCGTCGTGGTGATTG | AGAAGGTGGCAGTGGTAACCAG |
| *hSPP1* | CGAGGTGATAGTGTGGTTTATGG | GCACCATTCAACTCCTCGCTTTC |
| *hTCF21* | CACTTGAGGCAGATCCTGGCTA | CGGTCACCACTTCTTTCAGGTC |
| *mAdamts4* | GAACGGTGGCAAGTATTGTGAGG | TTCGGTGGTTGTAGGCAGCACA |
| *mActa2* | ACTCTCTTCCAGCCATCTTTCA | ATAGGTGGTTTCGTGGATGC |
| *mAger* | GCCACTGGAATTGTCGATGAGG | GCTGTGAGTTCAGAGGCAGGAT |
| *mCol1a1* | CCAAGAAGACATCCCTGAAGTCA | TGCACGTCATCGCACACA |
| *mCthrc1* | TGTTCAGGACCTCTTCCCATCG | GCCACATCTACCAATCCAGCAC |
| *mHprt* | CCTAAGATGAGCGCAAGTTGAA | CCACAGGACTAGAACACCTGCTAA |
| *mSftpc* | GGTCCTGATGGAGAGTCCAC | GATGAGAAGGCGTTTGAGGT |
| *mTimp3* | AGGATGCCTTCTGCAACTCCGA | GTGTAGACCAGAGTGCCAAAGG |

h: Human; m: Mouse

*ACTA2*: Actin alpha 2, smooth muscle

*ADAMTS4*: ADAM metallopeptidase with thrombospondin type 1 motif 4

*Ager*: Advanced glycosylation end-product specific receptor

*CEBPB*: CCAAT enhancer binding protein beta

*COL1A1*: Collagen type I alpha 1 chain

*CTHRC1*: Collagen triple helix repeat containing 1

*HOPX*: HOP homeobox

*Hprt*: Hypoxanthine guanine phosphoribosyl transferase

*INMT*: Indolethylamine N-methyltransferase

*LIMCH1*: LIM and calponin homology domains 1

*NPNT*: Nephronectin

*PBGD*: Porphobilinogen deaminase

*PDGFRA*: Platelet-derived growth factor receptor alpha

*PECAM1*: Platelet and endothelial cell adhesion molecule 1

*PLIN2*: Perilipin 2

*PPARG*: Peroxisome proliferator activated receptor gamma

*SFTPC*: Surfactant protein C

*SPP1*: Secreted phosphoprotein 1

*TCF21*: Transcription factor 21

*Timp3*: Tissue inhibitor of metalloproteinase 3
